# Supplementary material for: Heat adaptation of phage T7 under an extended genetic code
Source: Virus Evol. 2021 Dec 1;7(2):veab100. doi: 10.1093/ve/veab100 (PMC8923235; doi:10.1093/ve/veab100)
Supplement: veab100_Supp [file veab100_supp.zip › Sequencing_Analysis.zip!/Sequencing_Analysis/S2/evidence/JC_SIDE_1_42.html]

BRESEQ :: Evidence


| New junction evidence | | | | | | | | | | | |
| --- | --- | --- | --- | --- | --- | --- | --- | --- | --- | --- | --- |
|  | | seq id | position | reads (cov) | reads (cov) | score | skew | freq | annotation | gene | product |
| \* | ? | NC\_001604.1 | 295 = | 7072 (1.340) | 31 (0.010) | 13/560 | 48.9 | 0.47% | intergenic (–/–) | *–/–* | –/– |
|  | ? | NC\_001604.1 | = 281 | 6648 (1.350) |  |  |  |  | intergenic (–/–) | *–/–* | –/– |
| Rejected: Coverage evenness skew score above cutoff. | | | | | | | | | | | |
| Rejected: Frequency below/above cutoff threshold. | | | | | | | | | | | |

Only 100 of 7104 total aligned reads displayed.  

`‑‑‑‑‑TCTCACAGTGTACGGACCTAAAGTTCCCCCATAGGGGGTACCTAAAGCCCAGCCAATCACCTAAAGTCAACCTTCGGTTGACCTTGAGGGTTCCCTAAGGGTTGGGGATGACCCTTGGGTTTGTCTTTGGGTGTTACCTTGAGTGTCTCTCTGTGTCCCTATCTGTTACAGTCTCCTAAAGTATCCTCCTAAAGTCACCTCCTAACGTCCATCCTAAAGCCAACACCTAAAGCCTACACCTAAAGACCCATCAAGTCAACGCCTATCTTAAAGTTTAAACATAAAGACCAGACCTAAAGACCAGACCTAAAGAC‑ACTACATAAAGACCAGACCT‑AAAGACGCCTTGTTGTTAGCCATAAAGTGATAACCTTTAATCATTGTCTTTATTAATA‑CAACTCACTATAAGGAGAGACAACTTAAAGAGACTTAAAAGATTAATTTAAAATTTATCAAAA‑AGAGTATTGA‑CTTAAAGTCTAACCTATAGGATACTTACAGCCATCGAGAGGGACACGGCGAATAGCCATCCCAATCGACACCGGGGTCAACCGGATAAGTAGACAGCCTGATAAGTCGCACGAAAAACAGGTATTGACAA  >  NC_001604.1/1‑595`  

                                                                                                                                                                                                                                                                                                           `|`                                                                                                                                                                                                                                                                                                                   
`tgtataagagacaggtACGGACCTAAAGTTCCCCCATAGGGTGTACCTAAAGCCCAGCCAATCACCTAAAGTCAACCTTCGGTTGACCTTGAGGGTTCCCTAAGGGTTGGGGATGACCCTTGGGTTTGTCTTTGGGTGTTACCTTGAGTGTCTCTCTGTGTCCCTATCTGTTACAGTCTCCTAAAGTATCCTCCTAAAGTCACCTCCTAACGTCCATCCTAAAGCCAACACCTAAAGCCTACACCTAAAGACCCATCAAGTCAACGCCTATCTTAAAGTTTAAACAtaaagaccagaccta                                                                                                                                                                                                                                                                                                                  <  1:106944/287‑1 (MQ=255)`  
   `ataagagacaggtACGGACCTAAAGTTCCCCCATAGGGGGTACCTAAAGCCCAGCCAATCACCTAAAGTCAACCTTCGGTTGACCTTGAGGGTTCCCTAAGGGTTGGGGATGACCCTTGGGTTTGTCTTTGGGTGTTACCTTGAGTGTCTCTCTGTGTCCCTATCTGTTACAGTCTCCTAAAGTATCCTCCTAAAGTCACCTCCTAACGTCCATCCTAAAGCCAACACCTAAAGCCTACACCTAAAGACCCATCAAGTCAACGCCTATCTTAAAGTTTAAACAtaaagaccagacctaaag                                                                                                                                                                                                                                                                                                               <  1:659044/290‑1 (MQ=255)`  
     `gagacaggtgtACGGACCTAAAGTTCCCCCATAGTGGGTACCTAAATCCCAGCCAATCACCTAAAGTCAACCTTCGGTTGACCTTGAGGGTTCCCTAAGGGTTGGGGATGACCCTTGGGTTTGTCTTTGGGTGTTACCTTGAGTGTCTCTCTGTGTCCCGATCTGTTACAGTCTCCTAAAGTATCCTCCTAAAGTCACCTCCTAACGTCCATCCTAAAGCCAACACCTAAAGCCTACACCTAAAGACCCAACAAGTCAACGCCTATCTTAAAGTTTAAACAtaaagaccagacctaaagac                                                                                                                                                                                                                                                                                                             <  1:543656/294‑1 (MQ=255)`  
         `acaGTGTACGGACCTAAAGTTCCCCCATAGGGGGTACCTAAAGCCCAGCCAATCACCTAAAGTCAACCTTCGGTTGACCTTGAGGGTTCCCTAAGGGTTGGGGATGACCCTTGGGTTTGTCTTTGGGTGTTACCTTGAGTGTCTCTCTGTGTCCCTATCTGTTACAGTCTCCTAAAGTATCCTCCTAAAGTCACCTCCTAACGTCCATCCTAAAGCCAACACCTAAAGCATACACCTAAAGACCCATCAAGTCAACGCCTATCTTAAAGTTTAAACAtaaagaccagacctaaagaccaaa                                                                                                                                                                                                                                                                                                         >  1:356663/1‑299 (MQ=255)`  
          `caGTGTACGGACCTAAAGTTCCCCCATAGGGGGTACCTAAAGCCCAGCCAATCACCTAAAGTCAACCTTCGGTTGACCTTGAGGGTTCCCTAAGGGTTGGGGATGACCCTTGGGTTTGTCTTTGGGTGTTACCTTGAGTGTCTCTCTGTGTCCCTATCTGTTCCAGTCTCCTAAAGTATCCTCCTAAAGTCACCTCCTAACGTCCATCCTAAAGCCAACACCTAAAGCCTACACCTAAAGACCCATCAAGTCAACGCCTATCTTAAAGTTTAAACAtaaagaccagacctaaagaccagac                                                                                                                                                                                                                                                                                                        >  1:363629/1‑301 (MQ=255)`  
            `gtgtACGGACCTAAAGTTCCCCCATAGGGGGTACCTAAAGCCCAGCCAATCACCTAAAGTCAACCTTCGGTTGACCTTGAGGGTTCCCTAAGGGTTGGGGATGACCCTTGGGTTTGTCTTTGGGTGTTACCTTGAGTGTCTCTCTGTGTCCCTATCTGTTCCAGTCTCCTAAAGTATCCTCCTAAAGTCACCTCCTAACGTCCATCCTAAAGCCAACACCTAAAGCCTACACCTAAAGACCCATCAAGTCAACGCCTATCTTAAAGTTTAAACATaaagaccagacctaaagaccagacct                                                                                                                                                                                                                                                                                                      >  1:312297/1‑301 (MQ=255)`  
            `gtgtACGGACCTAAAGTTCCCCCATAGGGGGTACCTAAAGCCCAGCCAATCACCTAAAGTCAACCTTCGGTTGACCTTGAGGGTTCCCTAAGGGTTGGGGATGACCCTTGGGTTTGTCTTTGGGTGTTACCTTGAGTGTCTCTCTGTGTCCCTATCTGTTACAGTCTCCTAAAGTATCCTCCTAAAGTCACCTCCTAACGTCCATCCTAAAGCTAACACCTAAAGCCTACAACTAAAGACCCATCAAGTCAACGCCTATCTTAACGTTTAAACAtaaagaccagacctaaagcagtcacat                                                                                                                                                                                                                                                                                                      >  1:817566/1‑292 (MQ=255)`  
            `gtgtACGGACCTAAAGTTCCCCCATAGGGGGTACCTAAAGCCCAGCCAATCACCTAAAGTCAACCTTCGGTTGACCTTGAGGGTTCCCTAAGGGTTGGGGATGACCCTTGGGTTTGTCTTTGGGTGTTACCTTGAGTGTCTCTCTGTGTCCCTATCTGTTACAGTCTCCTAAAGTATCCTCCTAAAGTCACCTCCTAACGTCCATCCTAAAGCCAACACCTAAAGCCTACACCTAAAGACCCATCAAGTCAACGCCTATCTTAAAGTTTAAACAtaaagaccagacctaaagaccaaaact                                                                                                                                                                                                                                                                                                      >  1:678946/1‑296 (MQ=255)`  
            `gtgtACGGACCTAAAGTTCCCCCATAGGGGGTACCTAAAGCCCAGCCAATCACCTAAAGTCAACCTTCGGTTGACCTTGAGGGTTCCCTAAGGGTTGGGGATGACCCTTGGGTTTGTCTTTGGGTGTTACCTTGAGTGTCTCTCTGTGTCCCTATCTGTTACAGTCTCCTAAAGTATCCTCCTAAAGTCACCTCCTAACGTCCATCCTAAAGCCAACACCTAAAGCCTACACCTAAAGACCCATCAAGTCAACGCCTATCTTAAAGTTTAAACATaaagaccagacctaaagaccagacct                                                                                                                                                                                                                                                                                                      >  1:723331/1‑301 (MQ=255)`  
            `gtgtACGGACCTAAAGTTCCCCCATAGGGGGTACCTAAAGCCCAGCCAATCACCTAAAGTCAACCTTCGGTTGACCTTGAGGGTTCCCTAAGGGTTGGGGATGACCCTTGGGTTTGTCTTTGGGTGTTACCTTGAGTGTCTCTCTGTGTCCCTATCTGTTACAGTCTCCTAAAGTATCCTCCTAAAGTCACCTCCTAACGTCCATCCTAAAGCCAACACCTAAAGCCTACACCTAAAGACCCATCAAGTCAACGCCTATCTTAAAGTTTAAACATaaagaccagacctaaagaccagacct                                                                                                                                                                                                                                                                                                      >  1:643708/1‑301 (MQ=255)`  
            `gtgtACGGACCTAAAGTTCCCCCATAGGGGGTACCTAAAGCCCAGCCAATCACCTAAAGTCAACCTTCGGTTGACCTTGAGGGTTCCCTAAGGGTTGGGGATGACCCTTGGGTTTGTCGTTGGGTGTTACCTGAAGTGTCTCTCCGTGTCCCTATCTGTTACAGTCTCCTAAAGTAGCCTCCTAAAGTCACCTCCGAACGTCCATCCTAAAGCCAACACCTAAAGCCTACACCTAAAGACGCATCAAGTCAACGCCGATCTTAAAGTTTAAACAtaaagaccagaactctgactattatac                                                                                                                                                                                                                                                                                                      >  1:370359/1‑288 (MQ=25)`  
            `gtgtACGGACCTAAAGTTCCCCCATAGGGGGTACCTAAAGCACAGCCAATCACCTAAAGTCAACCTTCGGTTGACCTTGAGGGTTCCCTAAGGGTTGGGGATGACCCTTGGGTTTGTCTTTGGGTGTTACCTTGAGTGTCTCTCTGTGTCCCTATCTGTTACAGTCTCCTAAAGTATCCTCCTAAAGTCACCTCCTAACGTCCATCCTAAAGCCAACACCTAAAGCCTACACCTAAAGACCCATCAAGTCAACGCCTATCTTAAAGTTTAAACAtaaagaccagacctaaagctgtctctt                                                                                                                                                                                                                                                                                                      >  1:701821/1‑292 (MQ=255)`  
                   `gACCTAAAGTTCCCCCATAGGGGGTACCTAAAGCCCAGCCAATCACCTAAAGTCAACCTTCGGTTGACCTTGAGGGTTCCCTAAGGGTTGGGGATGACCCTTGGTTTTGTCTTTGGTTGTTACCTTGAGTGTCTCTCTGTGTCCCTATCTGTTACAGTCTCCTAAAGTATCCTCCTAAAGTCACCTCCTAACGTCCATCCTAAAGCCAACACCTAAAGCCTACACCTAAAGACCCATCAAGTCAACGCATATCTTAAAGTTTAAACATAAAGAACAGACCTAAAGACCAGAACTATAGac‑a                                                                                                                                                                                                                                                                                              >  1:566830/1‑301 (MQ=255)`  
                   `gACCTAAAGTTCCCCCATAGGGGGTACCTAAAGCCCAGCCAATCACCTAAAGTCAACCTTCGGTTGACCTTGAGGGTTCCCTAAGGGTTGGGGATGACCCTTGGGTTTGTCTTTGGGTGTTACCTTGAGTGTCTCTCTGTGTCCCTATCTGTTCCAGTCTCCTAAAGTATCCTCCTAAAGTCACCTCCTAACGTCCATCCTAAAGCCAACACCTAAAGCCTACACCTAAAGACCCATCAAGTCAACGCCTATCTTAAAGTTTAAACATAAAGACCAGACCTAAAGACCAGACCTAAAGac‑a                                                                                                                                                                                                                                                                                              >  1:271029/1‑301 (MQ=255)`  
                   `gACCTAAAGTTCCCCCATAGGGGGTACCTAAAGCCCAGCCAATCACCTAAAGTCAACCTTCGGTTGACCTTGAGGGTTCCCTAAGGGTTGGGGATGACCCTTGGGTTTGTCTTTGGGTGTTACCTTGAGTGTCTCTCTGTGTCCCTATCTGTTACAGTCTCCTAAAGTATCCTCCTAAAGTCACCTCCTAACGTCCATCCTAAAGCCAACACCTACAGCCTACACCTAAATACCCCTCAAGTCAACGCCTATCTTAAAGTTTAAACATAAAGACCAGACCTAAAGACCAGACCTAAAGac‑a                                                                                                                                                                                                                                                                                              >  1:319677/1‑301 (MQ=255)`  
                   `gACCTAAAGTTCCCCCATAGGGGGTACCTAAAGCCCAGCCAATCACCTAAAGTCAACCTTCGGTTGACCTTGAGGGTTCCCTAAGGGTTGGGGATGACCCTTGGGTTTGTCTTTGGGTGTTACCTTGAGTGTCTCTCTGTGTCCCTATCTGTTACAGTCTCCTAAAGTATCCTCCTAAAGTCACCTCCTAACGTCCATCCTAAAGCCAACACCTAAAGCCTACACCTAAAGACCCATCAAGTCAACGCCTATCTTAAAGTTTAAACAtaaagaccagacctgtctcttatacacatctccg                                                                                                                                                                                                                                                                                               >  1:620103/1‑281 (MQ=255)`  
                   `gACCTAAAGTTCCCCCATAGGGGGTACCTAAAGCCCAGCCAATCACCTAAAGTCAACCTTCGGTTGACCTTGAGGGTTCCCTAAGGGTTGGGGATGACCCTTGGGTTTGTCTTTGGGTGTTACCTTGAGTGTCTCTCTGTGTCCCTATCTGTTACAGTCTCCTAAAGTATCCTCCTAAAGTCACCTCCTAACGTCCATCCTAAAGCCAACACCTAAAGCCTACACCTAAAGACCCATCAAGTCAACGCCTATCTTAAAGTTTAAACATAAAGACCAGACCTAAAGACCAGACCTAAAGac‑a                                                                                                                                                                                                                                                                                              >  1:49847/1‑301 (MQ=255)`  
                   `gACCTAAAGTT‑CCCCATAGGGGGTACCTAAAGCCCAGCCAATCACCTAAAGTCAACCTTCGGTTGACCTTGAGGGTTCCCTAAGGGTTGGGGATGACCCTTGGGTTTGTCTTTGGGTGTTACCTTGAGTGTCTCTCTGTGTCCCTATCTGTTACAGTCTCCTAAAGTATCCTCCTAAAGTCACCTCCTAACGTCCATCCTAAAGCCAACACCTAAAGCCTACACCTAAAGACCCATCAAGTCAACGCCTATCTTAAAGTTTAAACATAAAGTCCAGACCTACAGACCAGACCTAAAGac‑ac                                                                                                                                                                                                                                                                                             >  1:428576/1‑301 (MQ=255)`  
                               `gcccATAGGGGGTACCTAAAGCCCAGCCAATCACCTAAAGTCAACCTTCGGTTGACCTTGAGGGTTCCCTAAGGGTTGGGGATGACCCTTGTGTTTGTCTTTGGGTGTTACCTTGAGTGTCTCTCTGTGTCCCTATCTGTTCCAGTCTCCTAAAGTATCCTCCTAAAGTCACCTCCTAACGTCCATCCTAAATCCAACACCTAAAGCCTACACCTAAAGACCCATCAAGTCAACGCCTATCTTAAAGTTTAAACATAAAGACCAGACCTAAAGACCAGACCTAAAGAC‑ACTACATAAagac                                                                                                                                                                                                                                                                                  <  1:185484/300‑1 (MQ=255)`  
                                          `gTACCTAAAGCCCAGCCAATCACCTAAAGTCAACCTTCGGTTGACCTTGAGGGTTCCCTAAGGGTTGGGGATGACCCTTGGGTTTGTCTTTGGGTGTTACCTTGAGTGTCTCTCTGTGGCCCTATCTGTTACAGTCTCCTAAAGTATACTTCTAAAGTCACCTCCTAACGTCCATCCTAAAGCCAACACTTAAAGCCTACACCTAAAGACCCATCAAGTCAACGCCTATCTTAAAGTTTAAACATAAAGACAAGACCTAAAGACCAGACCTAAAGAC‑ACTACATAAAGACCAGACTT‑AAAg                                                                                                                                                                                                                                                                      >  1:589647/1‑301 (MQ=255)`  
                                           `tACCTAAAGCCCAGCCAATCACCTAAAGTCAACCTTCGGTTGACCTTGAGGGTTCCCTAAGGGTTGGGGATGACCCTTGGGTTTGTCTTTGGGTGTTACCTTGAGTGTCTCTCTGTGTCCCTATCTGTTACAGTCTCCTAAAGTATCCTCCTAAAGTCACCTCCTAACGTCCATCCTAAAGCCAACACCTAAAGCCTACACCTAAAGACCCATCAAGTCAACGCCTATCTTAAAGTTTAAACATAAAGACCAGACCTAAAGACCAGACCTAAAGAC‑ACTACATAAagactgtctcttatac                                                                                                                                                                                                                                                                      >  1:278604/1‑289 (MQ=255)`  
                                            `aCCTAAAGCCCAGCCAATCACCTAAAGTCAACCTTCGGTTGACCTTGAGGGTTCCCTAAGGGTTGGGGATGACCCTTGGGTTTGTCTTTGGGTGTTACTTTGAGTGTCTCTCTGTGTCCCTATCTGTTACAGTCTCCTAAAGTATCCTCCTAAAGTCACCTCCTAACGTCCATCCTAATGCCAACACCTTAAGCCTACACCTAAATACCCATCAAGTCCACGCCTATCTTAAAGTTTAAATATAAAGACCAGACCTAAAGACCAGACTTAAAGAC‑CCTACATAAAGACCAGACCT‑AAAGAc                                                                                                                                                                                                                                                                    >  1:256323/1‑301 (MQ=255)`  
                                            `aCCTAAAGCCCAGCCAATCACCTAAAGTCAACCTTCGGTTGACCTTGAGGGTTCCCTAAGGGTTGGGGATGACCCTTGGGTTTGTCTTTGGGTGTTACCTTGAGTGTCTCTCTGTGTCCCTATCTGTTACAGTCTCCTAAAGTATCCTCCTAAAGTCACCTCCTAACGTCCATCCTAAAGCCAACACCTAAAGCCTACACCTAAAGACCCATCAAGTCAACGCCTATCTTAAAGTTTAAACATAAAGACCAGACCTAAAGACCAGACCTAAAGAC‑ACTACATAAAGACCAGACCT‑AAAGAc                                                                                                                                                                                                                                                                    >  1:206720/1‑301 (MQ=255)`  
                                            `aCCTAAAGCCCAGCCAATCACCTAAAGTCAACCTTCGGTTGACCTTGAGGGTTCCCTAAGGGTTGGGGATGACCCTTGGGTTTGTCTTTGGGTGTTACCTTGAGTGTCTCTCTGTGTCCCGATCTGTTACAGTCTCCTAAAGTATCCTCCTAAAGTCACCTCCTAACGTCCATCCTAAAGCCAACACCTAAAGCCTACACCTAAAGACCCATCAAGTCAACGCCTATCTTAAAGTTTAAACATAAAGACCAGACCTAAAGACCAGACCTAAAGAC‑ACTACAtctgtctcttatacacatct                                                                                                                                                                                                                                                                     >  1:249569/1‑282 (MQ=255)`  
                                                      `cAGCCAATCACCTAAAGTCAACCTTCGGTTGACCTTGAGGGTTCCCTAAGGGTTGGGGATGACCCTTGGGTTTGTCTTTGGGTGTTACCTTGAGTGTCTCTCTGTGTCCCTATCTGTTCCAGTCTCCTAAAGTATCCTCCTAAAGTCACCTCCTAACGTCCATCCTAAAGCCAACACCTAAAGCCTACACCTAAAGACCCATCAAGTCAACGCCTATCTTAAAGTTTAAACATAAAGACCAGACCTAAAGACCAGACCTAAAGAC‑ACTACATAAAGACCAGACCT‑AAAGACGCCTtgttgt                                                                                                                                                                                                                                                          >  1:581309/1‑301 (MQ=255)`  
                                                           `aaTCACCTAAAGTCAACCTTCGGTTGACCTTGAGGGTTCCCTAAGGGTTGGGGATGACCCTTGGGTTTGTCTTTGGGTGTTACCTTGAGTGTCTCTCTGTGTCCCTATCTGTTACAGTCTCCTAATGTATCCTCCTAAAGTCACCTACTAACGTCCATCCTAAAGCCAACACCTAAAGCCTACACCTAAAGACCCATCAAGTCAACGCCTATCTTAAAGTTTAAACATAAAGACCAGACCTAAAGACCAGACCTAAAGAC‑ACTACATAAAGACCAGACCT‑AAAGACGCCTTGTTGTTAGcc                                                                                                                                                                                                                                                     <  1:789794/301‑1 (MQ=255)`  
                                                                `ccTAAAGTCAACCTTCGGTTGACCTTGAGGGTTCCCTAAGGGTTGGGGATGACCCTTGGGTTTGTCTTTGGGTGTTACCTTGAGTGTCTCTCTGTGTCCCTATCTGTTACAGTCTCCTAAAGTATCCTCCTAAAGTCCCCTCCTAACGTCCATCCTAAAGCCAACACCTAAAGCCTACACCTAAAGACCCATCAAGTCAACGCCTATCTTAAAGTTTAAACATATAGACCAGACCTAAAGACCAGACCTAAAGCC‑ACTACATAAAGACCAGACCT‑AAAGACGCCTTGTTGTTAGCCATaaa                                                                                                                                                                                                                                                >  1:333845/1‑301 (MQ=255)`  
                                                                        `cAACCTTCGGTTGACCTTGAGGGTTCCCTAAGGGTTGGGGATGACCCTTGGGTTTGTCTTTGGGTGTTACCTTGAGTGTCTCTCTGTGT‑CCTATCTGTTACAGTCTCCTAAAGTATCCTCCTAAAGTCACCTCCTAACGTCCATCCTAAAGCCAACACCTAAAGCCTACACCTAAAGACCCATCAAGTCAACGCCTATCTTAAAGTTTAAACATAAAGACCAGAACTAAAGACCAGACCTAAAGAC‑ACTACATAAAGACCAGACCT‑AAAGACGCCTTGTTGTTAGCCATAAAGTGATAAcc                                                                                                                                                                                                                                       <  1:177226/301‑1 (MQ=255)`  
                                                                         `aaCCTTCTGTTGACCTTGAGTGTTCCCTAAGGGTTGGGTATGACCCTTGGGTTTGTCTTTTGGTGTTACCTTGAGTGTCTCTCTGTGTCCCTATCTGTTACAGTCTCCTAAAGTATCCTCCTAAAGTCACCTCCCAACGTCCATCCTAACGCCAACACCTAAAGCCTACACCTAAAGACCCATCAAGTCAACGCCTATCTTAAAGTTTAAACATAAAGACCAGACCTAAAGACCAGACCTAAAGAC‑ACTACATAAAGACCAGACCT‑AAAGACGCCTTGTTGTTAGCCATAAAGTGATAAcc                                                                                                                                                                                                                                       <  1:443769/301‑1 (MQ=255)`  
                                                                         `aaCCTTCGGTTGACCTTGAGGGTTCCCTAAGTGTTGGGGATGACCCTTGGGTTTGTCTTTGGGTGTTACCTTGAGTGTCTCTCTGTGTCCCGATCTGTTACAGTCTCCTAAAGTATCCTCCTAAAGTCACCTCCTAACGTCCATCCTAAAGCCAACACCTAAAGCCTACACCTAAAGACCCATCAAGTCAACGCCTATCTTAAAGTTTAAACATAAAGACCAGACCTAAAGACCAGACCTAAAGAC‑ACTACATAAAGACCAGACCT‑AAAGACGCCTTGTTGTTAGCCATAAAGTGATAAcc                                                                                                                                                                                                                                       <  1:651144/301‑1 (MQ=255)`  
                                                                              `tCGGTTGACCTTGAGGGTTCCCTAAGGGTTGGGGATGACCCTTGGGTTTGTCTTTGGGTGTTACCTTGAGTGTCTCTCTGTGTCCCGATCTGTTACAGTCTCCTAAAGTATCCTCCTAAAGTCACCTCCTAACGTCCATCCTAAAGCCAACACCTAAAGCCTACACCTAAAGACCCATCAAGTCAACGCCTATCTTAAAGTTTAAACATAAAGACCAGACCTAAAGACCAGACCTAAAGAC‑ACTACATAAAGACCAGACCT‑AAAGACGCCTTGTTGTTAGCCATAAAGTGATAAccctgtc                                                                                                                                                                                                                                  >  1:739772/1‑296 (MQ=255)`  
                                                                                `ggTTGACCTTGAGGGTTCCCTAAGGGTTGGGGATGACCCTTGGGTTTGTCTTTGGGTGTTACCTTGAGTGTCTCTCTGTGTCCCGATCTGTTACAGTCTCCTAAAGTATCCTCCTAAAGTCACCTCCTAACGTCCATCCTAAAGCCAACACCTAAAGCCTACACCTAAAGACCCATCAAGTCAACGCCTATCTTAAAGTTTAAACATAAAGACCAGACCTAAAGACCAGACCTAAAGAC‑ACTACATAAAGACCAGACCT‑AAAGACGCCTTGTTGTTAGCCATAAAGTGATAACCTTTAATc                                                                                                                                                                                                                                <  1:748339/301‑1 (MQ=255)`  
                                                                                    `gACCTTGAGGGTTCCCTAAGGGTTGGGGATGACCCTTGGGTTTGTCTTTGGGTGTTACCTTGAGTGTCTCTCTGTGTCCCTATCTGTTACAGTCTCCTAAAGTATCCTCCTAAAGTCACCTCCTAACGTCCATCCTAAAGCCAACACCTAAAGCCTACACCTAAAGACCCTTCAAGTCAACGCCTATCTTAAAGTTTAAACATAAAGACCAGACCTAAAGACCAGACCTAAAGAC‑ACTACATAAAGACCAGACCT‑AAAGACGCCTTGTTGTTAGCCATAAAGTGATAACCTTTAATcctgt                                                                                                                                                                                                                            >  1:457259/1‑297 (MQ=255)`  
                                                                                      `ccTTGAGGGTTCCCTAAGGGTTGGGGATGACCCTTGGGTTTGTCTTTTGGTGTTACCTTGAGTGTCTCTCTGTGTCCCTATCTGTTACAGTCTCCTAAAGTATCCTCCTAAAGTCACCTCCTAACGTCCATCCTAAAGCCAACACCTAAAGCCTACACCTAAAGACCCATCAAGTCAACGCCTATCTTAATGTTTAAACATAAAGACCAGACCTAAAGACCAGACCTAAAGAC‑ACTACATAAAGACCAGACCT‑AAAGACGCCTTGTTGTTAGCCATAAAGTGATAAccctgtctcttatac                                                                                                                                                                                                                          >  1:201835/1‑288 (MQ=255)`  
                                                                                      `ccTTGAGGGTTCCCTAAGGGTTGGGGATGACCCTTGGGTTTGTCTTTGGGTGTTACCTTGAGTGTCTCTCTGTGTCCCTATCTGTTACAGTCTCCTAAAGTATCCTCCTAAAGTCACCTCCTAACGTCCATCCTAAAGCCAACACCTAAAGCCTACACCTAAAGACCCATCAAGTCAACGCCTATCTTAAAGTTTAAACATAAAGACCAGACCTAAAGACCAGACCTAAAGAC‑ACTACATAAAGACCAGACCT‑AAAGACGCCTTGTTGTTAGCCATAAAGTGATAACCTTTAATCATTGTc                                                                                                                                                                                                                          <  1:437917/301‑1 (MQ=255)`  
                                                                                            `gacagcccTAAGGGTTGGGGATGACCCTTGGGTTTGTCTTTGGGTGTTACCTTGAGTGTCTCTCTGTGTCCCTATCTGTTACAGTCTCCTAAAGTATCCTCCTAAAGTCACCTCCTAACGTCCATCCTAAAGCCAACACCTAAAGCCTACACCTAAAGACCCATCAAGTCAACGCCTATCTTAAAGTTTAAACATAAAGACCAGACCTAAAGACCAGACCTAAAGAC‑ACTACATAAAGACCATACCT‑AAAGACGCCTTGTTGTTAGCCATAAAGTGATAACCTTTAATCATTGTCTttatt                                                                                                                                                                                                                    <  1:664400/296‑1 (MQ=255)`  
                                                                                                 `cccTAAGGGTTGGGGATGACCCTTGGGTTTGTCTTTGGGTGTTACCTTGAGTGTCTCTCTGTGTCCCTATCTGTTCCAGTCTCCTAAAGTATCCTCCTAAAGTCACCTCCTAACGTCCATCCTAAAGCCAACACCTAAAGCCTACACCTAAAGACCCATCAAGTCAACGCCTATCTTAAAGTTTAAACATAAAGACCAGACCTAAAGACCAGACCTAAAGAC‑ACTACATAAAGACCAGACCT‑AAAGACGCCTTGTTGTTAGCCATAAAGTGATAACCTTTAATCATTGTCTTTATTAATaa                                                                                                                                                                                                               >  1:709739/1‑300 (MQ=255)`  
                                                                                                 `cccTAAGGGTTGGGGATGACCCTTGGGTTTGTCTTTGGGTGTTACCTTGAGTGTCTCTCTGTGTCCCTATCTGTTCCAGTCTCCTAAAGTATCCTCCTAAAGTCACCTCCTAACGTCCATCCTAAAGCCAACACCTAAAGCCTACACCTAAAGACCCATCAAGTCAACGCCTATCTTAAAGTTTAAACATAAAGACCAGACCTAAAGACCAGACCTAAAGAC‑ACTACATAAAGACCAGACCT‑AAAGACGCCTTGTTGTTAGCCATAAAGTGATAACCTTTAATCATTGTCTTTATTAATA‑c                                                                                                                                                                                                              <  1:482764/301‑1 (MQ=255)`  
                                                                                                 `cccTAAGGGTTGGGGATGACCCTTGGGTTTGTCTTTGGGTGTTACCTTGAGTGTCTCTCTGTGTCCCTATCTGTTACAGTCTCCTAAAGTATCCTCCTAAAGTCACCTCCTAACGTCCATCCTAAAGCCAACACCTAAAGCCTACACCTAAAGACCCATCAAGTCAACGCCTATCTTAAAGTTTAAACATAAAGACCAGACCTAAAGACCAGACCTAAAGAC‑ACTACATAAAGACCAGACCT‑AAAGACGCCTTGTTGTTAGCCATAAAGTGATAACCTTTAATcctgtctcttatacacat                                                                                                                                                                                                               >  1:37850/1‑284 (MQ=255)`  
                                                                                                 `agacaggggTTGGGGATGACCCTTGGGTTTGTCTTTGGGTGTTACCTTGAGTGTCTCTCTGTGTCCCGATCTGTTACAGTCTCCTAAAGTATCCTCCTAAAGTCACCTCCTAACGTCCATCCTAAAGCCAACACCTAAAGCCTACACCTAAAGACCCATCAAGTCAACGCCTATCTTAAAGTTTAAACATAAAGACCAGACCTAAAGACCAGACCTAAAGAC‑ACTACATAAAGACCAGACCT‑GAAGACGCCTTGTTGTTAGCCATAAAGTGATAACCTTTAATCATTGTCTTTATTAATA‑c                                                                                                                                                                                                              <  1:115783/295‑1 (MQ=255)`  
                                                                                                  `ccTAAGGGTTGGGGATGACCCTTGGGTTTGTCTTTGGGTGTTACCTTGAGTGTCTCTCTGTGTCCCTATCTGTTCCAGTCTCCTAAAGTATCCTCCTAAAGTCACCTCCTAACGTCCATCCTAAAGCCAACACCTAAAGCCTACACCTAAAGACCCATCAAGTCAACGCCTATCTTAAAGTTTAAACATAAAGACCAGACCTAAAGACCAGACCTAAAGAC‑ACTACATAAAGACCAGACCT‑AAAGACGCCTTGTTGTTAGCCATAAAGTGATAACCTTTAATCATTGTCTTTATTAATA‑Ca                                                                                                                                                                                                             >  1:796505/1‑301 (MQ=255)`  
                                                                                                        `ggTTTGGGATGACCCTTGGGTTTGTCTTTGGGTGTTACCTTGAGTGTCTCTCTGTGTCCCTATCTGTTCCAGTCTCCTAAAGTATCCTCCTAAAGTCACCTCCTAACGTCCATCCTAAAGCCAACACCTAAAGCCTACACCTAAAGACCCATCAAGTCAACGCCTATCTTAAAGTTTAAACATAAAGACCAGACCTAAAGACCAGACCTAAAGAC‑ACTACATAAAGACCAGACCT‑AAAGACGCCTTGTTGTTAGCCATAAAGTGATAACCTTTAATCATTGTCTTTATTAATA‑CAACTCAc                                                                                                                                                                                                       >  1:422385/1‑301 (MQ=255)`  
                                                                                                        `ggTTGGGGATGACCCTTGGGTTTGTCTTTGGGTGTTACCTTGAGTGTCTCTCTGTGTCCCTATCTGTTACAGTCTCCTAAAGTATCCTCCTAAAGTCACCTCCTAATGTCCATCCTAAAGCCAACACCTAAAGCCTACACCTAAATACCCATCAAGTCAACGCCTATCTTAAAGTTTAAACATAAAGACCAGACCTAAAGACCAGACCTAAAGAC‑ACTACATAAAGACCAGACCT‑AAAGACGCCTTGTTGTTAGCCATAAAGTGATAACCTTTAATcctgtctcttatacacatctccgac                                                                                                                                                                                                        >  1:782028/1‑277 (MQ=255)`  
                                                                                                               `ctcggagatgtgtataagagacaggTGTTACCTTGAGTGTCTCTCTGTGTCCCGATCTGTTACAGTCTCCTAAAGTATCCTCCTAAAGTCACCTCCTAACGTCCATCCTAAAGCCAACACCTAAAGCCTACACCTAAAGACCCATCAAGTCAACGCCTATCTTAAAGTTTAAACATAAAGACCAGACCTAAAGACCAGACCTAAAGAC‑ACTACATAAAGACCAGACCT‑AAAGACGCCTTGTTGTTAGCCATAAAGTGATAACCTTTAATCATTGTCTTTATTAATA‑CAACTCACTATAAgg                                                                                                                                                                                                <  1:627489/278‑1 (MQ=255)`  
                                                                                                               `aatgtgtataagagacagcTTTGGGTGTTACCTTGAGTGTCTCTCTGTGTCCCTATCTGTTACAGTCTCCTAAAGTATCCTCCTAAAGTCACCTCCTAACGTCCATCCTAAAGCCAACACCTAAAGCCTACACCTAAAGACCCATCAAGTCAACGCCTATCTTAAAGTTTAAACATAAAGACCAGACCTAAAGACCAGACCTAAAGAC‑ACTACATAAAGACCAGACCT‑AAAGACGCCTTGTTGTTAGCCATAAAGTGATAACCTTTAATCATTGTCTTTATTAATA‑CAACTCACTATAAgg                                                                                                                                                                                                <  1:597298/283‑1 (MQ=255)`  
                                                                                                                `aTGACCCTTGGGTTTGTCTTTGGGTGTTACCTTGAGTGTCTCTCTGTGTCCCTATCTGTTCCAGTCTCCTAAAGTATCCTCCTAAAGTCACCTCCTAACGTCCATCCTAAAGCCAACACCTAAAGCCTACACCTAAAGACCCATCAAGTCAACGCCTATCTTAAAGTTTAAACATAAAGACCAGACCTAAAGACCAGACCTAAAGAC‑ACTACATAAAGACCAGACCT‑AAAGACGCCTTGTTGTTAGCCATAAAGTGATAACCTTTAATCATTGTCTTTATTAATA‑CAACTCACTATAAGga                                                                                                                                                                                               <  1:694443/301‑1 (MQ=255)`  
                                                                                                                `aTGACCCTGGGGTCTGTCTTTGGGTGTTACCTTGAGTGTCTCTCTGTGTCCCTATCTGTTACAGTCTCCTAAAGTATCCTCCTAAAGTCACCTCCTAACGTCCATCCTAAAGCCAACACCTAAAGCCTGCACCTAAAGACCCATCAAGTCAACGCCGATCTTAAAGTTTAAACATAAAGACCAGACCTAAAGACCAGACCTAAAGAC‑ACTACATAAAGACCAGACCT‑AAAGACGCCGTGTTGTTAGCCATAAAGAGATAACCTTTAATCATTGTCTTTATTAATA‑CAACTCACTATAAGga                                                                                                                                                                                               <  1:234350/301‑1 (MQ=255)`  
                                                                                                                   `aCCCTTGGGTTTGTCTTTGGGTGTTACCTTGAGTGTCTCTCTGTGTCCCGATCTGTTACAGTCTCCTAAAGTATCCTCCTAAAGTCACCTCCTAACGTCCATCCTAAAGCCAACACCTAAAGCCTACACCTAAAGACCCATCAAGTCAACGCCTATCTTAAAGTTTAAACATAAAGACCAGACCTAAAGACCAGACCTAAAGAC‑ACTACATAAAGACCAGACCT‑AAAGACGCCTTGTTGTTAGCCATAAAGTGATAACCTTTAATCATTGTCTTTATTAATA‑CAACTCACTATAAGgagag                                                                                                                                                                                            >  1:574403/1‑301 (MQ=255)`  
                                                                                                                    `cccTTGGGTTTGTCTTTGGGTGTTACCTTGAGTGTCTCTCTGTGTCCCTATCTGTTCCAGTCTCCTAAAGTATCCTCCTAAAGTCACCTCCTAACGTCCATCCTAAAGCCAACACCTAAAGCCTACACCTAAAGACCCATCAAGTCAACGCCTATCTTAAAGTTTAAACATAAAGACCAGACCTAAAGACCAGACCTAAAGAC‑ACTAAATAAAGACAAGACCT‑AAAGACGCCTTGTTGTTAGCCATAAAGTGATAACCTTTAATCATTGTCTTTATTAATA‑CAAATCAATATAAGaagaga                                                                                                                                                                                           >  1:327512/1‑301 (MQ=255)`  
                                                                                                                    `cccTTGGGTTTGTCTTTGGGTGTTACCTTGAGTGTCTCTCTGTGTCCCTATCTGTTACAGTCTCCTAAAGTATCCTCCTAAAGTCACCTCCTAACGTCCATCCTAAAGCCAACACCTAAAGCCTACACCTAAAGACCCATCAAGTCAACGCCTATCTTAAAGTTTAAACATAAAGACCAGACCTAAAGACCAGACCTAAAGAC‑ACTACATAAAGACCAGACCT‑AAAGACGCCTTGTTGTTAGCCATAAAGTGATAACCTTTAATCATTGTCTTTATTAATA‑CAACTCACTATAAGgagaga                                                                                                                                                                                           >  1:226971/1‑301 (MQ=255)`  
                                                                                                                    `cccTTGGGTTTGTCTTTGGGTGTTACCTTGAGTGTCTCTCTGTGTCCC‑ATCTGTTATAGTCTCCTAAAGTATCCTCCTAAAGTCACCTCCTAACGTCCATCCTAAAGCCAACACCTAAAGCCTACACCTAAAGACCCATCAAGTCAACGCCTATCTTAAAGTTTAAACATAAAGACCAGACCTAAAGACCAGACCTAAAGAC‑ACTACATAAAGACCAGACCT‑AAAGACTCCTTGTTGTTAGCCATAAAGTGATAACCTTTAATCATTGTCTTTATTAATA‑CAACTCACTATAAGgagctgt                                                                                                                                                                                          >  1:92923/1‑297 (MQ=255)`  
                                                                                                                               `gTCTTTGGGTGTTACCTTGAGTGTCTCTCTGTGTCCCTATCTGTTCCAGTCTCCTAAAGTATCCTCCTAAAGTCACCTCCTAACGTCCATCCTAAAGCCAACACCTAAAGCCTACACCTAAAGACCCATCAAGTCAACGCCTATCTTAAAGATTACACATAAAGACCAGACCTAAAGACCAGACCTAAAGAC‑ACTACATAAAGACCAGACCT‑AAAGAGGCCTTGTTGTTAGCCATAAAGTGATAACATTTAATCATTGTCTTTATTAATA‑AAACTCAATATAAGGAGAGACAACTTAAaga                                                                                                                                                                                >  1:169656/1‑301 (MQ=255)`  
                                                                                                                               `gTCTTTGGGTGTTACCTTGAGTGTCTCTCTGTGTCCCTATCTGTTACAGTCTCCTAAAGTATCCTCCTAAAGTCACCTCCTAACGTCCATCCTAAAGCCAACACCTAAAGCCTACACATAAAGACCCATCAAGTCAACGCCTATCTTAAAGTTTAAACATAAAGACCAGACCTAACGACCAGACCTAAAGAC‑ACTACATAAAGACCAGACCT‑AAAGACGCCTTGTTTTTAGCCATAAAGTGATAACCTTTAATCATTGTCTTTATTAATA‑CACCTCACTATAAGGAGAGACactgtatctt                                                                                                                                                                                >  1:213968/1‑292 (MQ=255)`  
                                                                                                                                `tCTTTGGGTGTTACCTTGATTGTCTCTCTGTGTCCCGATCTGTTACAGTCTCCTAAAGTATCCTCCTAAAGTCACCTCCTAACGTCCATCCTAAAGCCAACACCTAAAGCCTACACCTAAAGACCCATCAAGTCAACGCCTATCTTAAAGTTTAAACATAAAGACCAGACCTAAAGACCAGACCTAAAGAC‑ACTACATAAAGACCAGACCT‑AAAGACGCCTTGTTGTTAGCCATAAAGTGATAACCTTTAATCATTGTCTTTATTAATA‑CAACTCACTATAAGGAGAGACAACTTAAagag                                                                                                                                                                               <  1:521364/301‑1 (MQ=255)`  
                                                                                                                                `tCTTTGGGTGTTACCTTGAGTGTCTCTCTGTGTCCCTATCTGTTACAGTCTCCTAAAGTATCCTCCTAAAGTCACCTCCTAACGTCCATCCTAAAGCCAACACCTAAAGCCTACACCTAAAGACCCATCAAGTCAACGCCTATCTTAAAGTTTAAACATAAAGACCAGACCTAAAGACCAGACCTAAAGAC‑ACTACATAAAGACCAGACCT‑AAAGACGCCTTGTTGTTAGCCATAAAGTGATAACCTTTAATCATTGTCTTTATTAATA‑CAACTCACTATAAGGAGAGACAACTTAAagag                                                                                                                                                                               <  1:122440/301‑1 (MQ=255)`  
                                                                                                                                       `gtgtTACCTTGAGTGTCTCTCTGTGTCCCTATCTGTTACAGTCTCCTAAAGTATCCTCCTAAAGTCACCTCCTAACGTCCATCCTCAAGCCAACACCTAAAGCCTACACCTAAAGACCCATCAAGTCAACGCCTATCTTAAAGTTTAAACATAAAGACCAGACCTAAAGACCAGACCTAAAGCC‑ACTACATAAAGACCAGACCT‑AAAGACGCCTTGTTGTTAGCCATAAAGTGATAACCTTTAATCATTGTCTTTATTATTA‑CAACTCACtatacgaactgtctcttatacacatctccgc                                                                                                                                                                        >  1:392315/1‑274 (MQ=255)`  
                                                                                                                                         `gtTACCTTGAGTGTCTCTCTGTGTCCCTATCTGTTCCAGTCTCCTAAAGTATCCTCCTAAAGTCACCTCCTAACGTCCATCCTAAAGCCAACACCTAAAGCCTACACCTAAAGACCCATCAAGTCAACGCCTATCTTAAAGTTTAAACATAAAGACCAGACCTAAAGACCAGACCTAAAGAC‑ACTACATAAAGACCAGACCT‑AAAGACGCCTTGTTGTTAGCCATAAAGTGATAACCTTTAATCATTGTCTTTATTAATA‑CAACTCACTATAAGGAGAGACAACTTAAAGAGACTTaaaaa                                                                                                                                                                      >  1:512475/1‑300 (MQ=255)`  
                                                                                                                                           `gatgtgtataagagacaggtgtCCCTATCTGTTACAGTCTCCTAAAGTATCCTCCTAAAGTCACCTCCTAACGTCCATCCTAAAGCCAACACCTAAAGCCTACACCTAAAGACCCATCAAGTCAACGCCTATCTTAAAGTTTAAACATAAAGACCAGACCTAAAGACCAGACCTAAAGAC‑ACTACATAAAGACCAGACCT‑AAAGACGCCTTGTTGTTAGCCATAAAGTGATAACCTTTAATCATTGTCTTTATTAATA‑CAACTCACTATAAGGAGAGACAACTTAAAGAGACTTAAAAGAt                                                                                                                                                                    <  1:473141/283‑1 (MQ=255)`  
                                                                                                                                            `aCCTTGAGTGTCTCTCTGTGTCCCTATCTGTTACAGTCTCCTAAAGTATCCTCCTAAAGTCACCTCCTAACGTCCATCCTAAAGCCAACACCTAAAGCCTACACCTAAAGACCCATCAAGTCAACGCCTATCTTAAAGTTTAAACATAAAGACCAGACCTAAAGACCAGACCTAAAGAC‑ACTACATAAAGACCAGACCT‑AAAGACGCCTTGTTGTTAGCCATAAAGTGATAACCTTTAATCATTGTCTTTATTAATA‑CAACTCACTATAAGGAGAGACAACTTAAAGAGACTTAAAAGttt                                                                                                                                                                   >  1:803676/1‑301 (MQ=255)`  
                                                                                                                                               `atgtgtataagagacagtCCCTATCTGTTACAGTCTCCTAAAGTATCCTCCTAAAGTCACCTCCTAACGTCCATCCTAAAGCCAACACCTAAAGCCTACACCTAAAGACCCATCAAGTCAACGCCTATCTTAAAGTTTAAACATAAAGACCAGACC‑‑‑‑‑‑‑‑‑‑‑‑‑‑‑‑‑‑‑‑‑‑‑‑‑‑‑TAAAGACCAGACCT‑AAAGACGCCTTGTTGTTAGCCATAAAGTGATAACCTTTAATCATTGTCTTTATTAATA‑CAACTCACTATAAGGAGAGACAACTTAAAGAGACTTAAAAGATTAATTTAAAATTTATCAAAA‑AGAGTATTGa                                                                                                                                     <  1:284266/285‑1 (MQ=255)`  
                                                                                                                                                `tGAGTTTCTCTCTGTGTCCCGATCTGTTACAGTCTCCTAAAGTATCCTCCTAAAGTCACCTCCTAACGTCCATCCTAAAGCCAACACCTAAAGACTACACCTAAAGACACATCAAGTCAACGCCTATCTTAAAGATAAAACATAAAGACCAGACCTAAAGACCAGACCTAAAGAA‑ACTACATAAAGACGGGGCCT‑AAAGACGCGTTGTTGTTAGCCATAAAGTGATAACCGTTAATCATTGTCTTTATTAATA‑CAACTCACTATAAGGAGAGACAACTTAAAGAGACTTAAAAGATTAAtt                                                                                                                                                               <  1:76484/301‑1 (MQ=255)`  
                                                                                                                                                      `gctcggaaatgtgtataagagacaggTCTCCTAAAGTATCCTCCTAAAGTCACCTCCTAACGACCATCCTAAAGCCAACACCTAAAGCCTACACCTAAAGACCCATCAAGTCAACGCCTATCTTAAAGTTTAAACATAAAGACCAGACCTAAAGACCAGACCTAAAGAC‑ACTACATAAAGACCAGACCT‑AAAGACGCCTTGTTGTTAGCCATAAAGTGATAACCTTTAATCATTGTCTTTATTAATA‑CAACTCACTATAAGGAGAGACAACTTAAAGAGACTTAAAAGATTAATTTAAAAt                                                                                                                                                         <  1:263774/276‑1 (MQ=255)`  
                                                                                                                                                                `tCCCTATCTGTTACAGTCTCCTAAAGTATCCTCCTAAAGTCACCTCCTAACGTCCATCCTAAAGCCAACACCTAAAGGCTACACCTAAAGACCCATCAAGTCAACGCCTATCTTAAAGTTTAAACATAAAGACCAGACCTAAAGACCAGACCTAAAGAC‑ACTACATAAAGACCAGACCT‑AAAGACGCCTTGTTGTTAGCCATAAAGTGATAACCTTTAATCATTGTCTTTATTAATA‑CAACTCACTATAAGGAGAGACAACTTAAAGAGACTTAAAAGATTAATTTAAAATTTATCaaaa‑a                                                                                                                                              >  1:291560/1‑301 (MQ=255)`  
                                                                                                                                                                     `aTCTGTTACAGTCTCCTAAAGTATCCTCCTAAAGTCACCTCCTAACGTCCATCCTAAAGCCAACACCTAAAGCCTACACCTAAAGACCCATCAAGTCAACGCCTATCTTAAAGTTTAAACCTAAAGACCAGACCTAAAGACCAGACCTAAAGAC‑ACTACATAAAGACCAGACCT‑AAAGACGCCTTGTTGTTAGCCATAAAGTGATAACCTTTAATCATTGTCTTTATTAATA‑CAACTCACTATAAGGAGAGACAACTTAAAGAGACTTAAAAGATTAATTTAAAATTTATCAAAA‑AGAGTa                                                                                                                                         >  1:671563/1‑301 (MQ=255)`  
                                                                                                                                                                     `aTCTGTTACAGTCTCCTAAAGTATCCTCCTAAAGTCACCTCCTAACGTCCATCCTAAAGCCAACACCTAAAGCCTACACCTAAAGACCCATCAAGTCAACGCCTATCTTAAAGTTTAAACATAAAGACCAGACCTAAAGACCAGACCTAAAGAC‑ACTACATAAAGACCAGACCT‑AAAGACGCCTTGTTGTTAGCCATAAAGTGATAACCTTTAATCATTGTCTTTATTAATA‑CAACTCACTATAAGGAGAGACAACTTAAAGAGACTTAAAAGATTAATTTAAAATTTATCAAAA‑AGAGTa                                                                                                                                         >  1:464452/1‑301 (MQ=255)`  
                                                                                                                                                                     `aTCTGTTACAGTCTCCT‑AAGTATCCTCCTAAAGTCACCCCCTAACGTCTATCCTAAAGCCAACACCTAAAGCATACAGTTAAAGACCCATCAAGTTAACGCCTATCTTAAAGTTTAAACATAAAGACCAGACCTAAAGACCAGACCTAAAGAC‑ACTACATATAGACCAGACAT‑AAAGACACCTTGTTGTCAGCCATAAAGTGATAACTTTTAATCATTGTCTTTATTAATA‑CAACTCAATATAAGGAGAGATAATTTTAAGAGACTTAAAAGATTAATTTAAAATTTATCAAAA‑AGAGTAt                                                                                                                                        >  1:825167/1‑301 (MQ=255)`  
                                                                                                                                                                      `caggTTCCAGTCTCCTAAAGTATCCTCCTAAAGTCACCTCCTAACGTCCATCCTAAAGCCAACACCTAAAGCCTACACCTAAAGACCCATCAAGTCAACGCCTATCTTAAAGTTTAAACATAAAGACCAGACCTAAAGACCAGACCTAAAGAC‑ACTACATAAAGACCAGACCT‑AAAGACGCCTTGTTGTTAGCCATAAAGTGATAACCTTTAATCATTGTCTTTATTAATA‑CAACTCACTATAAGGAGAGACAACTTAAAGAGACTTAAAAGATTAATTTAAAATTTATCAAAA‑AGAGTAt                                                                                                                                        <  1:299203/298‑1 (MQ=255)`  
                                                                                                                                                                          `ttACAGTCTCCTAAAGTAGCCTCCTAAAGACACCTCCTAACGGCCATACTAAAGCCAACACCGAAAGCCTACACCTAAAGACCCATCAAGTCAACGCCTATCTTAAAGTTTAAACATAAAGACCAGACCTAAAGACCAGACCTAAAGAC‑ACTACATAAAGACCAGACCT‑AAAGACGCCTTGTTGTTAGCCAGAAAGTGATAACCTTTAATCATTGTCTTTATTAATA‑CAACTCACTATAAGGAGAGACAACTTAAAGAGACTTAAAAGATTAATTTAAAATTTATCAAAA‑AGAGTATTGA‑c                                                                                                                                   <  1:305513/301‑1 (MQ=255)`  
                                                                                                                                                                          `aagagacagccTAAAGTATCCTCCTAAAGTCACCTCCTAACGTCCATCCTAAAGCCAACACCTAAAGCCTACACCTAAAGACCCATCAAGTCAACGCCTATCTTAAAGTTTAAACATAAAGACCAGACCTAAAGACCAGACCTAAAGAC‑ACTACATAAAGACCAGACCT‑AAAGACGCCTTGTTGTTAGCCATAAAGTGATAACCTTTAATCATTGTCTTTATTAATA‑CAACTCACTATAAGGAGAGACAACTTAAAGAGACTTAAAAGATTAATTTAAAATTTATCAAAA‑AGAGTATTGA‑c                                                                                                                                   <  1:549776/292‑1 (MQ=255)`  
                                                                                                                                                                                `cctcCTAAAGTATCCTCCTAAAGTCACCTCCAAACGTCCATCCTAAAGCCAACAACTAAAGCCTACACCTAAAAACCCATCAAGTCAACGCCTATCTTAAAGTTTAAACATAAAGACCAGACCTAAAGACCAGACCTAAAGAC‑ACTACATAAAGACCAGACCT‑AAAGGCGCCTTGTTGTTAGACATAAAGTGATAACCTTTAATCATTGTCTTTATTAATA‑CGACTCACTATAAGGAGAGACAACTTAAAGAGACTTAAAAGATTAATTTAAAATTTATCAAAA‑AGAGTATTGA‑CTTAAAg                                                                                                                             <  1:400906/300‑1 (MQ=255)`  
                                                                                                                                                                                 `ctcCTAAAGTATCCTCCTAAAGTCACCTCCTAACGTCCATCCTAAAGCCAACACCTAAAGCCTACACCTAAAGACCCATCAAGTCAACGCCTATCTTAAAGTTTAAACATAAAGACCAGACCTAACGACCAGACCTACAGAC‑ACTACATAAAGACCAGACCT‑AAAGACGCCTTGTTGTTAGCCATAAAGTGATAACCTTTAATCATTGTCTTTATTAATA‑CAACTCACTATAAGGTGAGCCAACTTAAAGAGACTTAAAAGATTAATTTAAAATTTATCAAAA‑AGAGTATTGA‑CTTAAAGt                                                                                                                            >  1:347855/1‑301 (MQ=255)`  
                                                                                                                                                                                 `ctcCTAAAGTATCCTCCTAAAGTCACCTCCTAACGTCCATCCTAAAGCCAACACCTAAAGCCTACACCTAAAGACCCATCAAGTCAACGCCTATCTTAAAGTTTAAACATAAAGACCAGACCTAAAGACCAGACCTAAAGAC‑ACTACATAAAGACCAGACCT‑AAAGACGCCTTGTTGTTAGCCATAAAGTGATAACCTTTAATCATTGTCTTTATTAATA‑CAACTCACTATAAGGAGAGACAACTTAAAGAGACTTAAAAGATTAATTTAAAATTTATCAAAA‑AGAGTATTGA‑CTTAAAGt                                                                                                                            >  1:146340/1‑301 (MQ=255)`  
                                                                                                                                                                                   `ccTAAAGTATCCTCCTAAAGTCACCTCCTAACGTCCATCCTAAAGCCAACACCTAAAGCCTACACCTAAAGACCCATCAAGTCAACGCCTATCTTAAAGTTTAAACATAAAGACCAGACCTAAAGACCAGACCTAAAGAC‑ACTACATAAAGACCAGACCT‑AAAGACGCCTTGTTGTTAGCCATAAAGTGATAACCTTTAATCATTGTCTTTATTAATA‑CAACTCACTATAAGGAGAGACAACTTAAAGAGACTTAAAAGATTAATTTAAAATTTATCAAAA‑AGAGTATTGA‑CTTAAAGTCt                                                                                                                          >  1:193580/1‑301 (MQ=255)`  
                                                                                                                                                                                      `aaaGTATCCTCCTAAAGTCACCTCCTAACGTCCATCCTAAAGCCAACACCTAAAGCCTACACCTAAAGACCCATCAAGTCAACGCCTATCTTAAAGTTTAAACATAAAGACCAGACCTAAAGACCAGACCTAAAGAC‑ACTACATAAAGACCAGACCT‑AAAGACGCCTTGTTGTTAGCCATAAAGTGATAACCTTTAATCATTGTCTTTATTAATA‑CAACTCACTATAAGGAGAGACAACTTAAAGAGACTTAAAAGATTAATTTAAAATTTATCAAAA‑AGAGTATTGA‑CTTAAAGTCTAAc                                                                                                                       >  1:13831/1‑301 (MQ=255)`  
                                                                                                                                                                                          `tATCCTCCTAAAGTCACCTCCTAACGTCCATCCTAAAGCCAACACCTAAAGCCTACACCTAAAGACCCATCAAGTCAACGCCTATCTTAAAGTTTAAACATAAAGACCAGACCTAAAGACCAGACCTAAAGAC‑ACTACATAAAGACCAGACCT‑AAAGACGCCTTGTTGTTAGCCATAAAGTGATAACCTTTAATCATTGTCTTTATTAATA‑CAACTCACTATAAGGAGAGACAACTTAAAGAGACTTAAAAGATTAATTTAAAATTTATCAAAA‑AGAGTATTGA‑CTTAAAGTCTAACCtat                                                                                                                   <  1:731357/301‑1 (MQ=255)`  
                                                                                                                                                                                             `cctcctAAAGTCACCTCCTAACGTCCATCCTAAAGCCAACACCTAAAGCCTACACCTAAAGACCCATCAAGTCAACGCCTATCTTAAAGTTTAAACATAAAGACCAGACCTAAAGACCAGACCTAAAGAC‑ACTACATACAGACCAGACCT‑AAAGACGCCTTGTTGTTAGCCATAAAGTGATAACCTTTAATCATTGTCTTTATTAATA‑CAACTCACTATAAGGCGAGACAACTTAAAGAGACTTAAAAGATTAATTTAAAATTTATCAAAA‑AGAGTATTGA‑CTTAAAgctgtctcttatac                                                                                                                >  1:131067/1‑288 (MQ=255)`  
                                                                                                                                                                                             `cctcctAAAGTCACCTCCTAACGTCCATCCTAAAGCCAACACCTAAAGCCTACACCTAAAGACCCATCAAGTCAACGCCTATCTTAAAGTTTAAACATAAAGACCAGACCTAAAGACCAGACCTAAAGAC‑ACTACATAAAGACCAGACCT‑AAAGACGCCTTGTTGTTAGCCATAAAGTGATAACCTTTAATCATTGTCTTTATTAATA‑CAACTCACTATAAGGAGAGACAACTTAAAGAGACTTAAAAGATTAATTTAAAATTTATCAAAA‑AGAGTATTGA‑CTTAAAGTCTAACCTATAgg                                                                                                                <  1:161306/301‑1 (MQ=255)`  
                                                                                                                                                                                              `ctcctAAAGTCACCTCCTAACGTCCATCCTAAAGCCAACACCTAAAGCCTACACCTAAAGACCCATCAAGTCAACGCCTATCTTAAAGTTTAAACATAAAGACCAGACCTAAAGACCAGACCTAAAGAC‑ACTACATAAAGACCAGACCT‑AAAGACGCCTTGTTGTTAGCCATAAAGTGATAACCTTTAATCATTGTCTTTATTAATA‑CAACTCACTATAAGGAGAGACAACTTAAAGAGACTTAAAAGATTAATTTAAAATTTATCAAAA‑AGAGTATTGA‑CTTAAAGTCTAACCTATAGGa                                                                                                               >  1:385362/1‑301 (MQ=255)`  
                                                                                                                                                                                                 `ctAAAGTCACCTCCTAACGTCCATCCTAAAGCCAACACCTAAAGCCTACACCTAAAGACCCATCAAGTCAACGCCTATCTTAAAGTTTAAACATAAAGACCAGACCTAAAGACCAGACCTAAAGAC‑ACTACATAAAGACCAGACCT‑AAAGACGCCTTGTTGTTAGCCATAAAGTGATAACCTTTAATCATTGTCTTTATTAATA‑CAACTCACTATAAGGAGAGACAACTTAAAGAGACTTAAAAGATTAATTTAAAATTTATCAAAA‑AGAGTATTGA‑CTTAAAGTCTAACCTATAGGATAc                                                                                                            <  1:686687/301‑1 (MQ=255)`  
                                                                                                                                                                                                          `cctcctAACGTCCATCCTAAAGCCAACACCTAAAGCCTACACCTAAAGACCCATCAAGTCAACGCCTATCTTAAAGTTTAAACATAAAGACCAGACCTAAAGACCAGACCTAAAGAC‑ACTACATAAAGACCAGACCT‑AAAGACGCCTTGTTGTTAGCCATAAAGTGATAACCTTTAATCATTGTCTTTATTAATA‑CAACTCACTATAAGGAGAGACAACTTAAAGAGACTTAAAAGATTAATTTAAAATTTATCAAAA‑AGAGTATTGA‑CTTAAAGTCTAACCTATAGGATACTTAcctgtc                                                                                                   >  1:605663/1‑296 (MQ=255)`  
                                                                                                                                                                                                          `cctcctAACGTCCATCCTAAAGCCAACACCTAAAGCCTACACCTAAAGACCCATCAAGTCAACGCCTATCTTAAAGTTTAAACATAAAGACCAGACCTAAAGACCAGACCTAAAGAC‑ACTACATAAAGACCAGACCT‑AAAGACGCCTTGTTGTTAGCCATAAAGTGATAACCTTTAATCATTGTCTTTATTAATA‑CAACTCACTATAAGGAGAGACAACTTAAAGAGACTTAAAAGATTAATTTAAAATTTATCAAAA‑AGAGTATTGA‑CTTAAAGTCTAACCTATAGGATACTTACAGCCa                                                                                                   >  1:504551/1‑301 (MQ=255)`  
                                                                                                                                                                                                             `cctAACGTCCATCCTAAAGCCAACACCTAAAGCCTACACCTAAAGACCCATCAAGTCAACGCCTATCTTAAAGTTTAAACATAAAGACCAGACCTAAAGACCAGACCTAAAGAC‑ACTACATAAAGACCAGACCT‑AAAGACGCCTTGTTGTTAGCCATAAAGTGATAACCTTTAATCATTGTCTTTATTAATA‑CAACTCACTATAAGGAGAGACAACTTAAAGAGACTTAAAAGATTAATTTAAAATTTATCAAAA‑AGAGTATTGA‑CTTAAAGTCTAACCTATAGGATACTTACAGCCATCg                                                                                                >  1:773493/1‑301 (MQ=255)`  
                                                                                                                                                                                                                  `cGTCCATCCTAAAGCCAACACCTAAAGCCTACACCTAAAGACCCATCAAGTCAACGCCTATCTTAAAGTTTAAACATAAAGACCAGACCTAAAGACCAGACCTAAAGAC‑ACTACATAAAGACCAGACCT‑AAAGACGCCTTGTTGTTAGCCATAAAGTGATAACCTTTAATCATTGTCTTTATTAATA‑CAACTCACTATAAGGAGATACAACTTAAAGAGACTTAAAAGATTAATTTAAAATTTATCAAAA‑AGAGTATTGA‑CTTAAAGTCTAACCTATAGGATACTTACAGCCATCGAGAgg                                                                                           >  1:5294/1‑301 (MQ=255)`  
                                                                                                                                                                                                                   `gTCCATCCTAAAGCCAACACCTAAAGCCTACACCTAAAGACCCATCAAGTCAACGCCTATCTTAAAGTTTAAACATAAAGACCAGACCTAAAGACCAGACCTAAAGAC‑ACTACATAAAGACCAGACCT‑AAAGACGCCTTGTTGTTAGCCATAAAGTGATAACCTTTAATCATTGTCTTTATTAATA‑CAACTCACTATAAGGAGAGACAACTTAAAGAAACTTAAAAGATTAATTTAAAATTTATCAAAA‑AGAGTATTGA‑CTTAAAGTCTAACCTATAGGATACTTAcctgtctcttataca                                                                                          >  1:220826/1‑287 (MQ=255)`  
                                                                                                                                                                                                                           `caaaGACAACACCTAAAGCCTACACCTAAAGACCCATCAAGTCAACGCCTATCTTAAAGTTTAAACATAAAGACCAGACCTAAAGACCAGACCTAAAGAC‑ACTACATAAAGACCAGACCT‑AAAGACGCCTTGTTGTTAGCCATAAAGTGATAACCTTTAATCATTGTCTTTATTAATA‑CAACTCACTATAAGGAGAGACAACTTAAAGAGACTTAAAAGATTAATTTAAAATTTATCAAAA‑AGAGTATTGA‑CTTAAAGTCTAACCTATAGGATACTTACAGCCATCGAGAGGGACACGGCg                                                                                  <  1:636556/300‑1 (MQ=255)`  
                                                                                                                                                                                                                              `aGCCAACACCTAAAGCCTACACCTAAAGACCCATCAAGTCAACGCCTATCTTAAAGTTTAAACATAAAGACCAGACCTAAAGACCAGACCTAAAGAC‑ACTACATAAAGACCAGACCT‑AAAGACGCCTTGTTGTTAGCCATAAAGTGATAACCTTTAATCATTGTCTTTATTAATA‑CAACTCACTATAAGGAGAGACAACTTAAAGAGACTTAAAAGATTAATTTAAAATTTATCAAAC‑AGAGTATTGA‑CTTAAAGTCTAACCTATAGGATACTTACAGCCATCGAGAGGGACACGGCGAAt                                                                               >  1:81035/1‑301 (MQ=255)`  
                                                                                                                                                                                                                                `ccAACACCTAAAGCCTACAACAAAAGACCCATAAAGTCAACACCTATCTTAAAGTGTAAACATAAAGACCAGACCTAAAGACCAGACCGAAAGAC‑ACTACATAAAGACCAGGCCT‑AAAGACGCCGTGTTAATAGACATAAAGTGATAACCTTTAATCATTGTCATTATTAATA‑CAACGCACTATAAGGAGAGACAACTTAAAGAGACTTAAAAGATTAATTTAAAATTTATCAAAA‑AGAGTATTGA‑CTTAAAGTCTAACCTATAGGATACTTACAGCCATCGAGAGGGACACGGCGAATAg                                                                             <  1:415697/301‑1 (MQ=255)`  
                                                                                                                                                                                                                                    `cacCTAAAGCCTACACCTAAAGACCCATCAAGTCAACGCCTATCTTAAAGTTTAAACATAAAGACCAGACCTAAAGACCAGACCTAAAGAC‑ACTACATAAAGACCAGACCT‑AAGGACGCCTTGTTGTTAGCCATAAAGTGATAACCTTTAATCATTGTCTTTATTAATA‑CAACTCACTATAAGGAGAGACAACTTAAAGAGACTTAAAAGATTAATTTAAAATTTATCAAAA‑AGAGTATTGA‑CTTAAAGTCTAACCTATAGGATACTTACAGCCATCGAGAGGGACACGGCGAATAGCCAt                                                                         >  1:450387/1‑301 (MQ=255)`  
                                                                                                                                                                                                                                     `acCTAAAGCCTACACCTAAAGACCCATCAAGTCAACGCCTATCTTAAAGTTTAAACATAAAGACCAGACCTAAGAACCA‑ACCTTAAGACAATTACATAAA‑AACAGAACTCAAAGAGCCATTTTTTTAAGCAATAATTAGATATCCTTT‑ATCAATGTATTTACTAATACCAAATAAC‑ATAAGGGAAGTCAACTTAAAGAAAATTAAAAAGATAATTTAAAAATTAACAAAACAGA‑TATTTACCTAAAAGTCTAACTTATAGGTAATTTACACACACAGAGAGGAACAAGCCGAAtgccggct                                                                        >  1:84552/1‑294 (MQ=255)`  
                                                                                                                                                                                                                                      `ccTAAAGCCTACACCTAAAGACCCATCAAGTCAACGCCTATCTTAAAGTTTAAACATAAAGACCAGACCTAAAGACCAGACCTAAAGAC‑ACTACATAAAGACCAGACCT‑AAAGACGCCTTGTTGTTAGCCATAAAGTGATAACCTTTAATCATTGTCTTTATTAATA‑CAACTCACTATAAGGAGAGACAACTTAAAGAGACTTAAAAGATTAATTTAAAATTTATCAAAA‑AGAGTATTGA‑CTTAAAGTCTAACCTATAGGATACTTACAGCCATCGAGAGGGACACGGCGAATAGCCATcc                                                                       <  1:755920/301‑1 (MQ=255)`  
                                                                                                                                                                                                                                      `ccTAAAGCCTACACCTAAAGACCCATCAAGTCAACGCCTATCTTAAAGTTTAAACATAAAGACCAGACCTAAAGACCAGACCTAAAGAC‑ACTACATAAAGACCAGACCT‑AAAGACGCCTTGTTGTTAGCCATAAAGTAATAACCTTTAATCATTGTCTTTATTAATA‑CAACTCACTATAAGGAGAGACAACTTAAAGAGACTTAAAAGATTAATTTAAAATTTATCAAAA‑AGAGTATTGA‑CTTAAAGTCTAACCTATAGGATACTTACAGCCATcctgtctcttatacacatctccgagcc                                                                       >  1:340895/1‑275 (MQ=255)`  
                                                                                                                                                                                                                                                     `tAAAGACCCATCAAGTCAACGCCTATCTTAAAGTTTAAACATAAAGACCAGACCTAAAGACCAGACCTAAAGAC‑ACTACATAAAGACCAGACCT‑AAAGACGCCTTGTTGTTAGCCATAAAGTGATAACCTTTAATCATTGTCTTTATTAATA‑CAACTCACTATAAGGAGAGACAACTTAAAGAGACTTAAAAGATTAATTTAAAATTTATCAAAA‑AGAGTATTGA‑CTTAAAGTCTAACCTATAGGATACTTACAGCCATCGAGAGGGACACGGCGAATAGCCATCCCAATCGACACCgggg                                                        <  1:408260/301‑1 (MQ=255)`  
                                                                                                                                                                                                                                                           `ccccTCAAGTCCACGCCTATCTTAAAGGTTAAACATAAAGACCAGACCTAAAGACCAGACCTAAAGAC‑ACTACATAAAGACCAGACCT‑AAAGACGCCTTGTTGTTAGCCATAAAGTGATAACCTTTAATCATTGTCTTTATTAATA‑CAACTCACTATAAGGAGAGACAACTTAAAGAGACTTAAAAGATTAATTTAAAATTTATCAAAA‑AGAGTATTGA‑CTTAAAGTCTAACCTATAGGATACTTACAGCCATCGAGAGGGACACGGCGAATAGCCATCCCAATCGACACCGGGGTCAAcc                                                  <  1:612683/301‑1 (MQ=255)`  
                                                                                                                                                                                                                                                             `cATCAAGTCAACGCCTATCTTAAAGTTTAAACATAAAGACCAGACCTAAAGACCAGACCTAAAGAC‑ACTACATAAAGACCAGACCT‑AAAGACGCCTTGTTGTTAGCCATAAAGTGATAACCTTTAATCATTGTCTTTATTAATA‑CAACTCACTATAAGGAGAGACAACTTAAAGAGACTTAAAAGATTAATTTAAAATTTTTCAAAA‑AGAGTATTGA‑CTTAAAGTCTAACCTATAGGATACTTACAGCCATCGAGATGGACACGGCGAATAGACATCCCAATCGACACCGGGGTAAACCgg                                                >  1:240779/1‑301 (MQ=255)`  
                                                                                                                                                                                                                                                                   `gTCAACGCCTATCTTAAAGTTTAAACATAAAGACCAGACCTAAAGACCAGACCTAAAGAC‑ACTACATAAAGACCAGACCT‑AAAGACGCCTTGTTGTTAGCCATAAAGTGATAACCTTTAATCATTGTCTTTATTAATA‑CAACTCACTATAAGGAGAGACAACTTAAAGAGACTTAAAAGATTAATTTAAAATTTATCAAAA‑AGAGTATTGA‑CTTAAAGTCTAACCTATAGGATACTTACAGCCATCGAGAGGGACACGGCGAATAGCCATCCCAATCGACACCGGGGTCAAcctgtctctt                                          >  1:154872/1‑293 (MQ=255)`  
                                                                                                                                                                                                                                                                       `aCGCCTATCTTAAAGTTTAAACATAAAGACCAGACCTAAAGACCAGACCTAAAGAC‑ACTACATAAAGACCAGACCT‑AAAGACGCCTTGTTGTTAGCCATAAAGTGATAACCTTTAATCATTGTCTTTATTAATA‑CAACTCACTATAAGGAGAGACAACTTAAAGAGACTTAAAAGATTAATTTAAAATTTATCAAAA‑AGAGTATTGA‑CTTAAAGTCTAACCTATAGGATACTTACAGCCATCGAGAGGGACACGGCGAATAGCCATCCCAATCGACACCGGGGTCAACCGGATAAGTAGAc                                      >  1:717377/1‑301 (MQ=255)`  
                                                                                                                                                                                                                                                                                 `tAAAGTTTAAACATAAAGACCAGACCTAAAGACCAGACCTAAAGAC‑ACTACATAAAGACCAGACCT‑AAAGACGCCTTGTTGTTAGCCATAAAGTGATAACCTTTAATCATTGTCTTTATTAATA‑CAACTCACTATAAGGAGAGACAACTTAAAGAGACTTAAAAGATTAATTTAAAATTTATCAAAA‑AGAGTATTGA‑CTTAAAGTCTAACCTATAGGATACTTACAGCCATCGAGAGGGACACGGCGAATAGCCATCCCAATCGACACCGGGGTCAACCGGATAAGTAGACAGCCTGATaa                            <  1:489943/301‑1 (MQ=255)`  
                                                                                                                                                                                                                                                                                     `gTTTAAACATAAAGACCAGACCTAAAGACCAGACCTAAAGAC‑ACTACATAAAGACCAGACCT‑AAAGACGCCTTGTTGTTAGCCATAAAGTGATAACCTTTAATCATTGTCTTTATTAATA‑CAACTCAATATAAGGAGAGACAACTTAAAGAGACTTAAAAGATTAATTTAAAATTTATCAAAA‑AGAGTATTGA‑CTTAAAGTCTAACCTATAGGATACTTACAGCCATCGAGAGGGACACGGCGAATAGCCATCCCAATCGACACCGGGGTCAACCGGATAAGTAGActgtctcttatacac                        >  1:100040/1‑287 (MQ=255)`  
                                                                                                                                                                                                                                                                                                  `gacaggacctaaagaccagacctaaagac‑ACTACATAAAGACCAGACCT‑AAAGACGCCTTGTTGTTAGCCATAAAGTGATAACCTTTAATCATTGTCTTTATTAATA‑CAACTCACTATAAGGAGAGACAACTTAAAGAGACTTAAAAGATTAATTTAAAATTTATCAAAA‑AGAGTATTGA‑CTTAAAGTCTAACCTATAGGATACTTACAGCCATCGAGAGGGACACGGCGAATAGCCATCCCAATCGACACCGGGGTCAACCGGATAAGTAGACAGCCTGATAAGTCGCACGAAAAACAgg           <  1:536819/296‑1 (MQ=255)`  
                                                                                                                                                                                                                                                                                                       `gacctaaagaccagacctaaagac‑ACTACATAAAGACCAGACCT‑AAAGACGCCTTGTTGTTAGCCATAAAGTGATAACCTTTAATCATTGTCTTTATTAATA‑CAACTCACTATAAGGAGAGACAACTTAAAGAGACTTAAAAGATTAATTTAAAATTTATCAAAA‑AGAGTATTGA‑CTTAAAGTCTAACCTATAGGATACTTACAGCCATCGAGAGGGACACGGCGAATAGCCATCCCAATCGACACCGGGGTCAACCGGATAAGTAGACAGCCTGATAAGTCGCACGAAAAACAgctgtct      >  1:558272/1‑295 (MQ=255)`  

                                                                                                                                                                                                                                                                                                           `|`                                                                                                                                                                                                                                                                                                                   
`‑‑‑‑‑TCTCACAGTGTACGGACCTAAAGTTCCCCCATAGGGGGTACCTAAAGCCCAGCCAATCACCTAAAGTCAACCTTCGGTTGACCTTGAGGGTTCCCTAAGGGTTGGGGATGACCCTTGGGTTTGTCTTTGGGTGTTACCTTGAGTGTCTCTCTGTGTCCCTATCTGTTACAGTCTCCTAAAGTATCCTCCTAAAGTCACCTCCTAACGTCCATCCTAAAGCCAACACCTAAAGCCTACACCTAAAGACCCATCAAGTCAACGCCTATCTTAAAGTTTAAACATAAAGACCAGACCTAAAGACCAGACCTAAAGAC‑ACTACATAAAGACCAGACCT‑AAAGACGCCTTGTTGTTAGCCATAAAGTGATAACCTTTAATCATTGTCTTTATTAATA‑CAACTCACTATAAGGAGAGACAACTTAAAGAGACTTAAAAGATTAATTTAAAATTTATCAAAA‑AGAGTATTGA‑CTTAAAGTCTAACCTATAGGATACTTACAGCCATCGAGAGGGACACGGCGAATAGCCATCCCAATCGACACCGGGGTCAACCGGATAAGTAGACAGCCTGATAAGTCGCACGAAAAACAGGTATTGACAA  >  NC_001604.1/1‑595`

  

Alignment Legend

Aligned base mismatch/match (shaded by quality score): `ATCG/ATCG` `< 3 ≤` `ATCG/ATCG` `< 11 ≤` `ATCG/ATCG` `< 25 ≤` `ATCG/ATCG` `< 36 ≤` `ATCG/ATCG` `< 38 ≤` `ATCG/ATCG`

Unaligned base: `atcg`    Masked matching base: `atcg`    Alignment gap: `‑`    Deleted base: `‑`

Reads not counted as support for junction

read\_name Not counted due to insufficient overlap past the breakpoint.

read\_name Not counted due to not crossing MOB target site duplication.
